# The Cause of Periodicity in Intermittent Fever

**Published:** 1874-10

**Authors:** A. C. Edwards

**Affiliations:** Alabama


### THE CAUSE OF PERIODICITY IN INTERMITTENT FEVER.

BY A. C. EDWARDS, M.D., OF ALABAMA.

This is a subject that has engaged the attention of the medical profession since medicine has been cultivated as a science. Many and various have been the theories advanced in relation thereto each of which has had its votaries to laud it for a season, then it passes away to give place to the views of some other enthusiastic investigator. It is with diffidence that I presume to add another to the long list of theories on this intricate subject; but the fact that malarial fevers have periodic exacerbations is evident, and an explanation of that fact is the purport of this paper. My first inquiry will be, What is the cause of malarial fever? A few years ago, any educated physician would nave answered, *Marsh Miasmata;* but the onward march of scientific investigation has shown this answer to be but the scapegoat for professional ignorance. We will examine some of the changes that occur in the transition from a state of health to the condition known as intermittent fever. It is an admitted fact, that there are certain localities more favorable to the production of this form of disease than other localities are.

A person in health exposes himself to this deleterious locality, and while there receives into his system something that brings about certain changes. These changes are brought about so gradually as to be almost imperceptible. Observation has shown that such an individual will remain in a state of complete health until a certain day after exposure, which is almost uniformly on the seventh day. On that day he will feel a slight chilly sensation, perhaps followed by a slight fever. What is this great enemy which has been taken into the system, and has produced such fearful results? And what has been the *modus operandi* by which it has caused them? In answer to the first inquiry, I will say that the great cause of all this disturbance is an animalcule, or an immense collection of animalcules, and, as this is an admitted fact, established by the researches of modern physiologists, I shall not enter into any argument to sustain it. In answer to the second question, I shall advance some views that, perhaps, will meet opposition from some of my medical brethren. I cannot give into the views of a distinguished writer, that all these phenomena are caused by these animalcules exciting a ferment in the blood, but shall endeavor to account for them on natural principles.

I shall proceed first by assuming that these animalcules are subject to the same laws that govern all the animal creation. That in accordance with these laws they require food for their sustenance, suited to their condition. Finding themselves imprisoned in the system, nature demands sustenance, and they are forced to appropriate the material with which they are surrounded. The position I take is, that the blood of a healthy man contains a principle that they can appropriate to their nourishment, and that so long as they find proper nourishment they remain quiet and the system tolerates their presence. That when the material in the blood, suitable to their nourishment, becomes exhausted, then they must seek other nourishment. That they begin then to feed on the internal coat of the arteries, thus attacking the organic structure of the system. Nature then revolts at their presence, and summons all her powers to enforce their ejectment from her domain. The irritation of the internal coats of the arteries causes a determination of the blood from the surface to the internal regions of the body. The surface of the body being denuded ot the heat-given principle, becomes cold and we have all the symptoms of a chill. When the animalcules obtain sufficient nourishment to satisfy them, they cease their irritation; the blood resumes its wonted channels, nature rouses herself to repair the injury, and we have all the phenomena of a fever succeeding a chill. The second position I take is, that there is a seven day's supply of food in the blood of a healthy person for these animalcules. Hence we find the system tolerates them for seven days, and until they have exhausted the supply of food in the blood and begin to attack the organic structure. A physician comes to the sick man and, after proper preparation, saturates the blood of the patient with quinine. Now is not the principle consumed by these animalcules from the blood of a healthy man identical with quinine? They, finding the blood saturated with a principle suited to their nourishment, cease their irritation to the arteries. Hence, in common parlance, the chill is broken. All physicians have marked the great proneness to a return of the chill on the seventh day; and why? Because, being but a seven days' supply of food furnished them, they have exhausted it and again attack the organic structure. A few observations as to the cause of the daily periodicity, and I am done. My view is, that they have stated times to seek their nourishment; that when that time rolls around, they begin their irritation and we have a chill; on the next day, at the same time, they must have food, and hence we have a recurrence of the paroxysm.

